# New pathogen, same disparities: why COVID‐19 and HIV remain prevalent in U.S. communities of colour and implications for ending the HIV epidemic

**DOI:** 10.1002/jia2.25639

**Published:** 2020-11-01

**Authors:** Gregorio A Millett

**Affiliations:** ^1^ amfAR Foundation for AIDS Research Washington DC USA

**Keywords:** disparities, COVID‐19, ending HIV epidemic, social determinants of health, structural racism

## Abstract

**Introduction:**

The U.S. Ending the HIV Epidemic (EHE) Initiative was launched nationally in February 2019. With a target of ending the HIV epidemic by 2030, EHE initially scales up effective HIV prevention and care in 57 localities that comprise the greatest proportion of annual HIV diagnoses in the United States (US). However, the EHE effort has been eclipsed by another infectious disease 11 months into the Initiative’s implementation. SARS‐COV‐2, a novel coronavirus, has infected more than eight million Americans and at least 223 000 (as of 23 October 2020) have succumbed to the disease. This commentary explores the social conditions that place communities of colour at greater risk for COVID‐19 and HIV, and assesses challenges to EHE in a post‐COVID‐19 universe.

**Discussion:**

One of the many common threads between HIV and COVID‐19 is the disproportionate impact of each disease among communities of colour. A recent report by the National Academy of Sciences surmised that as much as 70% of health outcomes are due to health access, socio‐economic factors and environmental conditions. Social determinants of health associated with greater HIV burden in Black and Brown communities have re‐emerged in epidemiological studies of disproportionate COVID‐19 cases, hospitalizations and deaths in communities of colour. Using data from the scientific literature, this commentary makes direct comparisons between HIV and COVID‐19 racial disparities across the social determinants of health. Furthermore, I examine three sets of challenges facing EHE: (1) Challenges that hamper both the EHE and COVID‐19 response (i.e. insufficiently addressing the social determinants of health; amplification of disparities as new health technologies are introduced) (2) Challenges posed by COVID‐19 (i.e. diverting HIV resources to address COVID‐19 and tapering of EHE funding generally); and (3) Challenges unrelated to COVID‐19 (i.e. emergence of new and related health disparities; repeal of the Affordable Care Act and long‐term viability of EHE).

**Conclusions:**

Racism and discrimination place communities of colour at greater risk for COVID‐19 as well as HIV. Achieving and sustaining an end to the U.S. HIV epidemic will require structural change to eliminate conditions that give rise to and maintain disparities.

## 1 INTRODUCTION

The United States (US) Ending the HIV Epidemic (EHE) Initiative was launched nationally in February 2019 [1]. The Initiative scales proven prevention and treatment interventions to reduce HIV transmission in localities and populations at high risk for HIV. Eleven months into the EHE initiative, the first case of SARS‐COV‐2 was diagnosed in the US [2]. Racial disparities in COVID‐19 diagnoses and deaths became apparent soon after the first reported cases.

As early as mid‐April, 2020, 22% of US counties with greater than a thirteen percent population of Black residents (the national average) accounted for 52% of COVID‐19 diagnoses and 57% of deaths nationwide [3]. As striking as these numbers are, the disproportionate impact of COVID‐19 has not only affected Black communities. The Centers for Disease Control and Prevention (CDC) reported that American Indians were 3.5 times more likely to be diagnosed with COVID compared to Whites [4]. Another analysis found that 75% of COVID‐19 ‘hot spot’ counties were disproportionately Latinx [5], whereas a separate study reported disproportionate COVID‐19 hospitalizations among people of colour across 12 states [6]. Although similar reports of racial disparities in COVID‐19 infections have been reported in France [7], the United Kingdom [8], Canada [9] and Brazil [10], the issue has been covered more exhaustively in the scientific and grey literature in the US.

This commentary fleshes out data presented during the opening plenary of the 2020 International AIDS Conference where similarities between COVID‐19 and HIV racial disparities in the US were investigated [11]. I also explore challenges facing the successful implementation of the EHE and implications for HIV racial disparities.

## 2 DISCUSSION

### 2.1 COVID‐19, HIV and social determinants of health

The COVID‐19 pandemic is actually a syndemic [12, 13]. A syndemic (or overlapping multiple epidemics) is “two or more afflictions, interacting synergistically, contributing to excess burden of disease in a population” [14]. The fact that at least three concurrent epidemics (COVID‐19, drug use, and HIV pandemics) disproportionately impact communities of colour in the US is not a chance occurrence. Systemic racism not “race” is at the root of these disparities. Systemic (or structural) racism may be defined as “A system in which public policies, institutional practices, cultural representations, and other norms work in various, often reinforcing ways to perpetuate racial group inequity” [15].

A spate of high‐profile police killings of Black Americans in tandem with the ongoing disproportionate impact of COVID‐19 by race and ethnicity catapulted structural racism into the popular lexicon. Black Lives Matter protests galvanized nationwide during the summer of 2020 in the wake of the murder of George Floyd [16]. Mr. Floyd’s death at the hands of police officers and his COVID‐19 diagnosis (revealed after his autopsy), crystallized for the US public the intersection between syndemics and structural racism [17].

Syndemics and structural racism may also explain why infectious disease burden remains concentrated in communities of colour despite greater reported preventative behaviours. For instance Black Americans remain more likely to acquire HIV despite greater HIV testing and fewer risk behaviours than White Americans [18, 19]. The same is true for COVID‐19. Although Black Americans are more likely than Whites to report wearing protective gear to prevent COVID‐19 transmission [20] or to social distance [21], the COVID‐19 pandemic continues to disproportionately impact Black communities.

**Figure 1:**
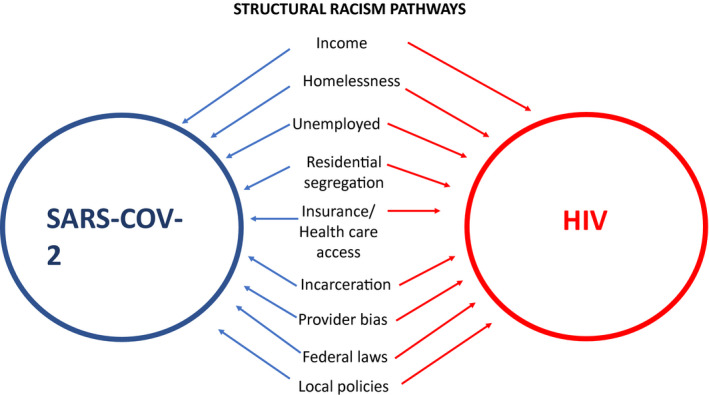
Structural racism operates through social determinants of health to exacerbate SARS‐COV‐2 and HIV disparities in communities of colour.

A recent National Academy of Sciences report suggests that as much as 70% of health outcomes stem from socio‐economic factors, physical environment, as well as health care [22]. Reviews of structural racism catalogue the contributions of these social determinants on poorer health outcomes in communities of colour [23, 24, 25]. Using economic, epidemiological and social science research, I summarize a subset of social determinants and their contribution to racial disparities in SARS‐COV‐2 and HIV infection. (see Figure 1).

#### 2.1.1 Income

Neither wealth nor income is concentrated in communities of colour in the US [26], and having a low income is associated with greater exposure to HIV and COVID‐19 infection. A study of 80 metropolitan US cities reported that poverty, unemployment and vacant housing were each associated with greater HIV incidence in Black communities [27]. Likewise, an analysis of US counties found that counties with a higher proportion of Latinos living in poverty experienced greater HIV disparities [28]. The COVID‐19 economic recession has only exacerbated poverty rates in Black and Brown communities. Communities of colour are bearing the brunt of the economic recession brought upon by the pandemic as reflected in statistics of those out of work [29] or at risk of eviction or foreclosure [30]. Moreover, Blacks and Latinos are more likely to have jobs that place them at greater risk for COVID‐19 exposure [31]. A recent analysis estimated that 65% of Latinx households and 57% of Black households had at least one person who could not work remotely during lockdown as compared to 47% of White households [32].

Racial and ethnic health disparities persist even among groups with high incomes. Gopal, et al (2013) found higher all‐cause HIV mortality rates among Blacks with higher incomes compared to their White counterparts [33]. Similarly, COVID‐19 infection and death rates in racially diverse counties with high incomes are three and four times (respectively) the rate of primarily White counties with comparably high incomes [34].

#### 2.1.2 Housing, Homelessness and Residential Segregation

Both substandard and lack of housing are associated with a panoply of health conditions [35], and communities of colour in the US are more likely to experience poorer housing conditions [36]. A San Francisco‐based cross‐sectional study of 1222 people living with HIV found a direct relationship between high viral load and being homeless [37]. Another study reported that people diagnosed with HIV while homeless have a 27‐fold greater odds of death compared to those diagnosed with access to shelter [38].

Housing conditions also create an environment that facilitates COVID‐19 transmission. Denser living quarters, specifically the number of residents per room, is associated with greater COVID‐19 diagnoses in epidemiological studies of Black [3] and Latinx [39] communities; and poor housing has also been implicated in COVID‐19 transmission among Native American communities [4].

**Figure 2:**
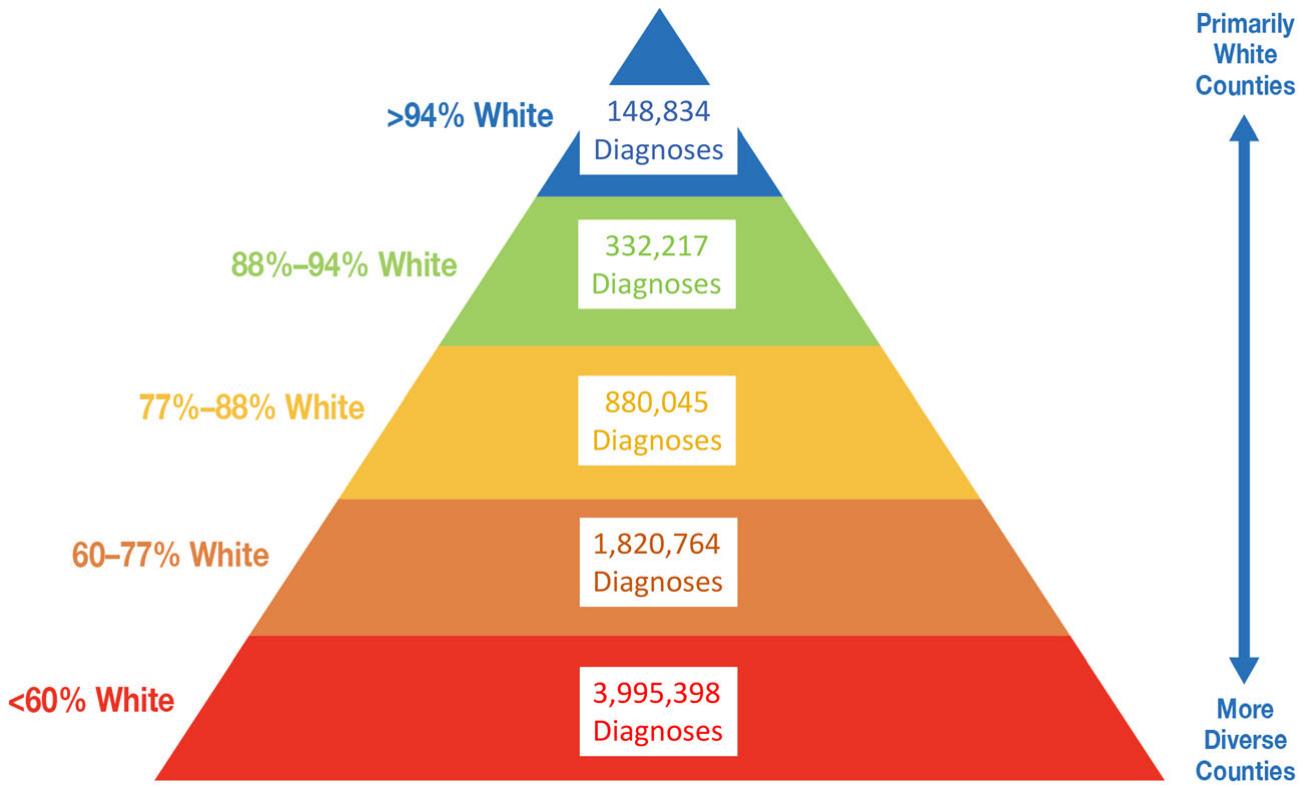
COVID‐19 diagnoses by proportion of White non‐Hispanic residents in US counties through September 30, 2020 (Source: https://ehe.amfar.org/inequity).

Where people live also determines health. The federal government’s promotion of racial segregation via redlining practices in the 1930s have had profound health effects that remain to this day [40]. Cities with greater residential segregation have higher HIV incidence [27]. The same associations by race/ethnicity have been reported for COVID‐19 cases and mortality [41]. Another analysis underscored the health risks associated with segregation by demonstrating that predominantly White (>88% residents) counties have had fewer COVID‐19 diagnoses than more diverse (<60% White residents) counties over the course of the US pandemic [42] (See Figure 2).

Meaningfully addressing racial segregation could improve health equity. An economic analysis reported that “a one standard deviation decrease in segregation would eliminate one‐third of the Black‐White differences” [43].

#### 2.1.3 Incarceration/detention centers

The United States has the highest rate of incarceration globally [44]. Harsher sentencing for the same offences [45], as well as a greater likelihood to be held in detention due to inability to post bail [46], place Blacks and Latinos at greater risk of imprisonment than White Americans [47]. These biases continue unabated in the COVID‐19 era. Nationwide reports show that law enforcement disproportionately target people of colour for violating mask mandates or social distancing guidelines [48, 49, 50].

Although the probability of transmission is lower than in the general population, HIV transmission has been documented in carceral settings [51]; and an estimated one in seven people living with HIV cycle through the prison system annually [52]. Incarceration is also associated with interruptions in HIV treatment during and after detention [53], which increases poor health outcomes for people living with HIV as well as the likelihood of HIV transmission.

Each of the top 10 COVID‐19 infection outbreaks nationwide have links to correctional facilities, and as many as 87 of the top 100 outbreaks are linked to detention centres [54]. Juvenile detention centres are not immune. Nearly 2000 cases of COVID‐19 have been documented in juvenile detention centres with 64% of residents (all Black) at one facility testing positive for COVID‐19 [55]. Because detention centres are more likely to be situated in communities of colour, they also contribute to health disparities via community spread of COVID‐19. A recent Chicago study linked community spread of COVID‐19 in Black communities to individuals passing through the county jail [56].

Immigration detention centres may also create environments that facilitate COVID‐19. As early as May, 2020, at least 5000 detainees nationwide had already tested positive for COVID‐19 [57]. Although Immigration and Customs Enforcement (ICE) do not test most detainees, details of a major outbreak at a Virginia facility emerged where more than 80% percent of detainees tested positive for COVID‐19 [58].

#### 2.1.4 Health care access

President Obama’s Affordable Care Act (ACA) increased healthcare access for an additional 20 million Americans, including people living with HIV [59, 60]. A component of the ACA, if adopted at the state level, extends healthcare access for low‐income Americans through the Medicaid programme. According to the Kaiser Family Foundation, the adoption of Medicaid by all states alone could insure an additional 15% of all Black Americans nationally [61]. Unfortunately, not all states have elected to expand Medicaid. As many as 60 000 people living with HIV live in states that have not expanded Medicaid, which exacerbates HIV disparities because Black Americans living with HIV are more likely to be insured by Medicaid than their White counterparts [62, 63]. Medicaid expansion could also improve healthcare for communities of colour who are more likely to be essential workers. A report by the Center on Budget and Policy Priorities announced that 650 000 uninsured essential workers could gain health coverage if the remaining hold out states expanded Medicaid [64].

The health benefits of Medicaid expansion are clear. It is associated with greater uptake of pre‐exposure prophylaxis (PrEP) [65], higher rates of continuous viral suppression [66] and declines in HIV incidence [67]. Moreover, greater insurance rates are associated with fewer COVID‐19 diagnoses [3]. Despite these and many other studies showing the benefits of Medicaid expansion, it has remained unpopular in more conservative states. The political ground is slowly shifting in favor of Medicaid expansion in light of the worsening dual health and economic crises prompted by COVID‐19, which may benefit the well‐being of Native, Black and Latinx communities (assuming the ACA survives any further legal challenges) [68].

### 2.2 New and existing challenges in ending the US HIV epidemic

The implementation of the EHE was already a challenge prior to COVID‐19 [69], but the pandemic has brought several challenges to the fore and has become a challenge itself to implementing EHE. Broadly speaking, challenges facing EHE can be categorized as: (1) similar to those obstructing the COVID‐19 response, (2) complicated by the COVID‐19 pandemic, or (3) unrelated to COVID‐19.

#### 2.2.1 Similar challenges facing EHE and the COVID‐19 response

##### Insufficiently targeting the social determinants of health

An estimated eight million Americans (disproportionately Black and Brown) have slipped into poverty since May 2020 [70]. People at risk for or living with HIV likely have a fundamentally different baseline of economic, housing and insurance stability in the wake of the COVID‐19 pandemic. In spite of these challenges, the EHE’s locus of implementation activities are primarily limited to the Department of Health and Human Services and presents a lost opportunity to mount a more aggressive response against the social determinants of health by marshalling resources across multiple departments in the federal government (e.g. Department of Justice, Department of Labor, Department of Housing and Urban Development, Department of Veterans Affairs).

The influence of the social determinants loom large among those affected by HIV or COVID‐19. A CDC study reported that HIV mortality was lower for people living with HIV in states with greater healthcare coverage, anti‐discrimination laws, and viral suppression among those enrolled in the Ryan White programme [71]. Likewise, an analysis of 11 210 COVID‐19 patients at 92 US hospitals found that racial disparities abated after adjusting for sociodemographic and clinical factors [72]; and a UK study reported that the greatest reduction in risk of COVID‐19 hospitalizations among Blacks versus Whites was the addition of socio‐economic factors (i.e. neighbourhood deprivation and household crowding) into the multivariable model [73].

Suitable EHE and COVID‐19 responses must be coordinated, government‐wide and part of a comprehensive plan with short‐ and long‐term targets that include the social determinants of health.

##### New health innovations will magnify racial disparities

Any new health innovations introduced during the EHE effort will magnify health disparities. The rollout of antiretroviral therapy and later PrEP were both marred by unequal access to these innovations by communities of colour [74, 75]. Likewise, COVID‐19 testing centres have been primarily located in White rather than Black or Brown communities across the US [76]. Long‐acting injectables may follow the same pattern, as well as other HIV innovations in the research and development pipeline. Similar access issues may complicate the rollout of the new Abbott BinaxNOW COVID‐19 rapid tests and the eventual rollout of the first COVID‐19 vaccines. History and experience indicate that these and other new innovations will widen both HIV and COVID‐19 racial disparities.

#### 2.2.2 Challenges the COVID‐19 pandemic poses for successfully implementing EHE

##### Funding for EHE may diminish

A major impediment to the successful implementation and sustainability of EHE is funding. Nearly $35 million was allocated to launch EHE in 2019, and $266 million was appropriated in 2020 with additional funding allocated through the Coronavirus Aids, Relief and Economic Security (CARES) Act. Although it is tacitly understood that EHE funding must increase in successive years to reach the Initiative’s 2030 targets, funding has fallen short. As of the writing of this Commentary, the House of Representatives only appropriated an additional $87 million in funding for EHE—far short of the $716 million FY’21 funds proposed by the Administration [77].

Funding to fight COVID‐19 will likely affect funding for other initiatives like the EHE. The Congressional Budget Office estimates that it will cost the U.S. $9 trillion dollars over the next 10 years to address the COVID‐19 crisis [78]. Larry Summers, former National Economic Council director in the Obama administration, places the estimate closer to $16 trillion [79]. Both estimates are devastating and could effectively squeeze out necessary funding for EHE and other health and social programmes.

##### HIV research and service disruption will magnify disparities

The COVID‐19 pandemic is delaying HIV research activities and affecting HIV care delivery. Clinical trials and lab research have yet to return to pre‐pandemic operations [80]. Moreover, HIV researchers in academic centres, federal health agencies, and health departments are playing double duty addressing HIV and COVID‐19 or have switched temporarily to COVID‐19 work entirely, which may slow EHE progress. There are also reports of HIV service disruption, particularly PrEP refill lapses and HIV testing during the economic shutdown [81]. Given a nationwide rise in COVID‐19 cases during the 2020 fall and winter season, future shutdowns will produce further delays in HIV research and care.

Ongoing COVID‐19 disruptions will extend the time horizon for ending HIV far beyond EHE’s 2030 target. A modelling analysis performed *before* the COVID‐19 pandemic found that four of six US cities will achieve HIV epidemic control by 2040 at the earliest [82]. Even more concerning, the study found that the time horizon for ending HIV in each of the cities will be years longer for Black and Latinx residents than Whites. Given COVID‐19 related disruptions in HIV prevention and care, the timeline for ending HIV in communities of colour may be considerably longer than previously estimated.

#### 2.2.3 Challenges to implementing EHE unrelated to COVID‐19

##### Emergence of new disparities

Unanticipated health challenges may present additional hurdles to implementing EHE. For instance, Black Americans are less likely to be prescribed opioids by providers [83]. This partially explains greater opioid overdoses among Whites versus Blacks nationwide. However, some disparities are fluid and not static. Reviews and meta‐analyses through the early 2000s repeatedly found that Black gay men were less likely than White gay men to use crystal methamphetamine (a highly addictive drug associated with HIV seroconversion) [20, 84]. But today use of crystal methamphetamine has not only increased markedly among Black gay men, but in some localities eclipses drug use among White gay men [85]. The same reversal may take place with the opioid epidemic and the Black community at large [86, 87]. EHE planning must be nimble enough to spot and address emerging health issues that may affect racial and ethnic disparities.

##### Repeal of the affordable care Act and EHE

Changes in the composition of the Supreme Court may imperil the Affordable Care Act and, in turn, EHE [88]. Expansion of access to health insurance is central to ending the HIV epidemic. The number of insured Americans increased nationally and across EHE phase I jurisdictions between 2012 and 2017 [89]. PrEP coverage is also higher in EHE jurisdictions that have expanded Medicaid compared [89]. Repeal of the ACA will have dire consequences for people at risk for or living with HIV. Moreover, elements of the law crucial for people living with or at risk for HIV will be lost such as: (1) the inability to discriminate against insuring people with pre‐existing health conditions; (2) coverage of reproductive health services for women; (3) non‐discrimination of LGBT patient by providers or (4) congressional oversight requiring each administration to provide biennial reports on addressing racial disparities in health.

## 3 CONCLUSIONS

How wonderful it is that nobody need wait a single moment before starting to improve the world. ‐Anne Frank

Racial and ethnic disparities in COVID‐19 are part of a predictable pattern of disease burden in the US that stem from structural racism. Although expansion of ACA and innovative models of health equity can reduce observed disparities, they may not be sufficient to end them [90, 91]. A sobering analysis published in PNAS asserted that COVID‐19 mortality among Whites in the US must increase by a factor of six to match the mortality rates among Blacks in any given year *prior* to the COVID‐19 pandemic [92].

For EHE to be successful and sustainable in racial and ethnic communities at greater risk for HIV, we must address the structural issues at the root of HIV and other health disparities. This requires a Marshall Plan that tackles inequities across health and other sectors, and creates economic, housing, educational opportunities for Black and Brown communities. It also requires re‐prioritization of the US budget to place a premium on primary as well as secondary prevention investments in communities of colour. Last, it requires meaningful representation in policy‐making bodies nationally and locally. An analysis found that various health policies consistently disadvantage communities of colour [93] likely due to under‐representation in Congress as well as the municipal level [94, 95]. This tension will become increasingly inexorable as demographics of the US change by 2044. Thankfully, we need not wait a moment longer to improve our world.

## COMPETING INTERESTS

None declared.

## AUTHOR CONTRIBUTIONS

GM devised and wrote the manuscript based upon his opening plenary for AIDS 2020.
